# Autoantigen microarrays reveal autoantibodies associated with proliferative nephritis and active disease in pediatric systemic lupus erythematosus

**DOI:** 10.1186/s13075-015-0682-6

**Published:** 2015-06-17

**Authors:** D. James Haddon, Vivian K. Diep, Jordan V. Price, Cindy Limb, Paul J. Utz, Imelda Balboni

**Affiliations:** Department of Medicine, Division of Immunology and Rheumatology, Stanford University School of Medicine, 291 Campus Drive, Stanford, CA 94305 USA; Department of Pediatrics, Division of Allergy, Immunology and Rheumatology, Stanford University School of Medicine, 291 Campus Drive, Stanford, CA 94305 USA; Department of Molecular and Cell Biology, Division of Immunology and Pathogenesis, University of California at Berkeley, 142 Life Sciences Addition #3200, Berkeley, CA 94720 USA

## Abstract

**Introduction:**

Pediatric systemic lupus erythematosus (pSLE) patients often initially present with more active and severe disease than adults, including a higher frequency of lupus nephritis. Specific autoantibodies, including anti-C1q, anti-DNA and anti-alpha-actinin, have been associated with kidney involvement in SLE, and DNA antibodies are capable of initiating early-stage lupus nephritis in severe combined immunodeficiency (SCID) mice. Over 100 different autoantibodies have been described in SLE patients, highlighting the need for comprehensive autoantibody profiling. Knowledge of the antibodies associated with pSLE and proliferative nephritis will increase the understanding of SLE pathogenesis, and may aid in monitoring patients for renal flare.

**Methods:**

We used autoantigen microarrays composed of 140 recombinant or purified antigens to compare the serum autoantibody profiles of new-onset pSLE patients (n = 45) to healthy controls (n = 17). We also compared pSLE patients with biopsy-confirmed class III or IV proliferative nephritis (n = 23) and without significant renal involvement (n = 18). We performed ELISA with selected autoantigens to validate the microarray findings. We created a multiple logistic regression model, based on the ELISA and clinical information, to predict whether a patient had proliferative nephritis, and used a validation cohort (n = 23) and longitudinal samples (88 patient visits) to test its accuracy.

**Results:**

Fifty autoantibodies were at significantly higher levels in the sera of pSLE patients compared to healthy controls, including anti-B cell-activating factor (BAFF). High levels of anti-BAFF were associated with active disease. Thirteen serum autoantibodies were present at significantly higher levels in pSLE patients with proliferative nephritis than those without, and we confirmed five autoantigens (dsDNA, C1q, collagens IV and X and aggrecan) by ELISA. Our model, based on ELISA measurements and clinical variables, correctly identified patients with proliferative nephritis with 91 % accuracy.

**Conclusions:**

Autoantigen microarrays are an ideal platform for identifying autoantibodies associated with both pSLE and specific clinical manifestations of pSLE. Using multiple regression analysis to integrate autoantibody and clinical data permits accurate prediction of clinical manifestations with complex etiologies in pSLE.

**Electronic supplementary material:**

The online version of this article (doi:10.1186/s13075-015-0682-6) contains supplementary material, which is available to authorized users.

## Introduction

Systemic lupus erythematosus (SLE) is a complex, chronic autoimmune disease with diverse signs and symptoms that commonly affect multiple organs and tissues. SLE has an unpredictable course, with periods of flares and remissions. High-titer autoantibodies targeting nuclear antigens, including DNA, RNA, histones and ribonucleoproteins (RNP), are a defining feature of SLE. Prior to diagnosis with SLE, patients gradually accumulate new autoantibodies, and have an average of three (from Ro, La, antiphospholipid (APL), antinuclear antibody (ANA), dsDNA, Smith, and RNP) at diagnosis [1]. Many patients likely have additional autoantibodies, as >100 autoantigens have been described in SLE [2]. Levels of autoantibodies fluctuate with disease activity and are associated with specific organ involvement in SLE [3]. Autoantibodies can directly cause pathology in SLE, as a human anti-DNA monoclonal antibody was capable of initiating early-stage lupus nephritis (LN) in severe combined immunodeficiency (SCID) mice [4].

Ten to twenty percent of SLE patients have disease onset in childhood or adolescence. Pediatric SLE (pSLE) patients often initially present with more acute and severe disease than adults [5], including a higher frequency of LN observed at presentation [6, 7]. LN is one of the primary causes of morbidity and mortality in pSLE [8]. Clinicians regularly evaluate urinary parameters, including hematuria, pyuria, cellular casts and proteinuria, to aid in the diagnosis and monitoring of LN. However, these metrics have low accuracy, especially in the context of monitoring for renal flare [9]. Candidate biomarkers for LN in pSLE include antibodies against dsDNA [3, 10], complement C3 and C4 levels [10], urine mRNAs [11], urine chemokines [12, 13], and urine proteins/peptides [14, 15]. While measurement of anti-dsDNA and complement C3 and C4 levels are commonly available clinical laboratory tests, only 50 % of LN patients display a decrease in C3 and C4 or increase in anti-dsDNA antibodies concurrent with a flare [9, 16]. While multiple factors influence the development of LN, including complement, autoantibodies, environment, and genetics [17], the majority of these approaches only measure single analytes, and may not capture the clinical heterogeneity in SLE.

Autoantigen microarrays allow highly multiplexed measurement of serum autoantibodies that recognize purified or recombinant protein and nucleic acid-containing autoantigens. Our group has developed microarrays to measure autoantibodies targeting known autoantigens [18, 19], cytokines and chemokines [20], and modified peptides [21]. This platform enables the characterization of multiple autoantibodies in parallel, while using microliter amounts of patient sera. To our knowledge, autoantigen microarrays have yet to be used to identify autoantibodies associated with pSLE or predictive of pSLE LN. An advantage of using highly multiplexed experimental platforms is that they can be used to identify multianalyte signatures or scores associated with clinical features of SLE. For example, gene expression microarrays were used to identify the interferon (IFN) signature, associated with active and severe forms of SLE, and protein microarrays were used to establish the chemokine score, associated with disease activity and predictive of flares in SLE [22–25].

In-depth knowledge of the diverse profiles of autoantibodies present in the serum of pSLE patients will increase our understanding of SLE, and aid in disease diagnosis and prognosis. There is significant interest in identifying autoantibody profiles that are associated with LN and predictive of renal flares, with a goal to enable preemptive treatment. In the current study, we utilized autoantigen microarrays to identify novel serum autoantibodies associated with pSLE. This included anti-B cell-activating factor (BAFF), which we found was associated with active disease. We also identified autoantibodies associated with proliferative nephritis in pSLE, including autoantibodies against aggrecan and collagens IV and X. Using autoantibody measurements in combination with clinical information, we developed a nephritis score that identified patients who have proliferative nephritis with high accuracy. In the future, a similar score could be used to enhance the diagnosis and monitoring of proliferative nephritis in pSLE patients.

## Methods

### Patients

SLE patients with presentation prior to age 18 who met American College of Rheumatology revised criteria for classification of SLE [26] were recruited at the Pediatric Rheumatology Clinic at Stanford Children’s Health and Lucile Packard Children’s Hospital Stanford. Age-appropriate consent and assent were obtained. Serum was collected from 70 new-onset patients and age- and sex-matched healthy control serum was purchased from Biodesign International Inc. (Saco, ME, USA) (n = 17). The Stanford University Institutional Review Board approved this study (IRB protocol number 13952). Patient demographics and characteristics at time of collection are available in Table 1. The patients were followed an average of 4.2 years (range 0–10.7 years). Pathologists classified patient kidney biopsies according to the International Society of Nephrology/Renal Pathology Society (ISN/RPS) 2003 classification [27]. The patient cohort was divided into training (n = 41) and test (n = 23) sets based on the order in which they were collected. The training set consists of pSLE patients with biopsy-confirmed class III or IV proliferative nephritis (n = 23), and patients with no significant evidence of proliferative nephritis (n = 18). Four patients were suspected to have nephritis, but were excluded from the training set and treated as ‘unknown’ because of inconclusive biopsy (n = 1), or lack of biopsy due to bleeding risk (n = 2) or cardiac involvement (n = 1). The nephrologist concluded that the first patient’s biopsy was inconclusive due to the presence of scar tissue, which prevented classification. We utilized a test set of 23 patients with (n = 8) biopsy-confirmed class III or IV proliferative nephritis, or with either biopsy-confirmed class II nephritis or without significant renal involvement (n = 15), to test accuracy of the prediction model. Two patients with class V nephritis were assayed alongside the test set to assess the model’s performance with membranous nephritis samples. Five pSLE patients from the test set were selected based on their disease progression for longitudinal analysis: two who developed biopsy-confirmed LN, at 351 days (class III) and 1303 days (class IV) after their initial visit, two age-matched controls who were biopsied and found to have class II LN, and a final subject who had a relatively stable disease course. Anti-dsDNA and anti-C1q immunoglobulin G (IgG) levels of each sample were determined by ELISA, and a chart review was performed to collect all applicable clinical data.

**Table 1:** Patient demographics

Variable	Training (n = 41)	Test (n = 23)	Combined (n = 64^a^)
Age at diagnosis, mean (range) years	13.9 (7.5–18.1)	13.7 (9.5–18.1)	13.8 (7.5–18.1)
Sampling date, postdiagnosis (range) days	15 (0–62)	5 (0–32)	12 (0–62)
Number (%) females	37 (90)	20 (87)	57 (89)
ANA-positive patients (%)	40 (98)	23 (100)	63 (98)
dsDNA antibody-positive patients (%)	35 (83)	19 (83)	53 (83)
Class III/IV lupus nephritis-biopsy proven^b^ (%)	23 (56)	8 (35)	31 (48)
Mean (range) SLEDAI score	14 (4–30)	14 (4–29)	14 (4–30)
Treatment at sample:			
PO steroids (%)	17 (41)	4 (20)	21 (33)
IV steroids (%)	18 (44)	1 (5)	19 (30)
HCQ (%)	18 (44)	8 (40)	26 (41)
Other IS (%)	6 (15)	0 (0)	6 (9)
None	10 (24)	12 (60)	22 (34)
Hispanic (%)	16 (39)	9 (39)	25 (39)
Asian/Pacific Islander (%)	12 (29)	8 (35)	20 (31)
Non-Hispanic Caucasian (%)	10 (24)	5 (22)	15 (23)
Black (%)	2 (5)	1 (4)	3 (5)

*ANA* antinuclear antibody, *SLEDAI* systemic lupus erythematosus disease activity index, *PO* per os, *IV* intravenous, *HCQ* hydroxychloroquine, *IS* immunosuppressive therapy (cyclophosphamide, mycophenolate, or methotrexate), *None* no treatment or NSAID alone

^a^Four new-onset patients who were suspected to have proliferative nephritis, but were not biopsy-confirmed, were not included in the training and test sets, or these demographics. Similarly, two patients with class V nephritis were not included in the training and test sets, or these demographics

^b^One patient who developed class IV nephritis 4 years after her initial visit, and a second patient who developed class V nephritis 5 years after her initial visit, were considered negative for this analysis

### Autoantigen microarrays

As previously described, we used autoantigen microarrays to identify patient serum autoantibodies [28]. Briefly, 140 recombinant or purified autoantigens were printed on the surface of 1-pad nitrocellulose FAST microarray slides (Whatman, GE Healthcare Life Sciences, Piscataway, NJ, USA), in replicates of six, using a VersArray ChipWriter Pro arraying robot (Bio-Rad Laboratories, Inc., Hercules, CA, USA). Silicon quill pins with 75 micron tips and approximately 120 nL reservoir volume were used (Parallel Synthesis Technologies, Santa Clara, CA, USA). Autoantigens were diluted in phosphate-buffered saline (PBS) at a concentration of 200 μg/mL (see Table S2 in Additional file 1 for a list of autoantigens). Microarrays were probed with patient serum, diluted 1/200 in phosphate-buffered saline with Tween 20 (PBST) with 3 % fetal calf serum (FCS), followed by detection using a Cy5-labeled goat anti-human IgG (Fcγ specific) secondary antibody (The Jackson Laboratory, Bar Harbor, ME, USA). Microarray slides were scanned using a GenePix 4000B Microarray Scanner (Molecular Devices, Sunnyvale, CA, USA). Fluorescent images were analyzed using GenePix Pro 6.0 Acquisition and Analysis Microarray Software (Molecular Devices). Median fluorescence intensity (MFI) minus background was determined for each feature. Negative MFI minus background signals were set to zero. The data discussed in this publication have been deposited in NCBI’s Gene Expression Omnibus [29] and are accessible through GEO Series accession number GSE69662 [30].

### Enzyme-linked immunosorbent assay (ELISA)

We performed serum autoantibody ELISA with selected autoantigens to validate the microarray findings. Ninety-six-well MaxiSorp ELISA plates (Nunclon, Thermo Fisher Scientific, Waltham, MA, USA) were coated with 2 ug/ml of the following antigens in PBS: complement C1q (Biodesign), histone H1, histones H2A and H4, histone H2B (Immunovision, Springdale, AR, USA), collagen types IV and X from human placenta (Sigma-Aldrich, St Louis, MO, USA), plasmid dsDNA (Diarect, Freiburg, Germany), aggrecan from bovine articular cartilage (Sigma-Aldrich), or bovine serum albumin (BSA, Sigma-Aldrich) as a negative control. Serum samples were diluted between 1/200 and 1/800 in PBS (with 0.05 % Tween-20 + 5 % FCS) and used to probe duplicate wells. Wells were incubated with dissociation-enhanced lanthanide fluoroimmunoassay (DELFIA) europium-labeled anti-human IgG (Fcγ specific; Perkin Elmer, Waltham, MA, USA), and a Wallac Victor model 1420 Multilabel Counter was used to measure time-resolved fluorescence (Perkin Elmer). The BSA signal of each serum sample was subtracted from the signal for a given antigen. The B cell-activating factor (BAFF) ELISA was performed according to a previously described protocol [20].

### Statistical analysis

Significance analysis of microarrays (SAM) was used to identify autoantigens with significantly different reactivity between pSLE patients and controls, and patients with and without proliferative nephritis [31]. Calculations were performed with R 3.0.2 [32] and the samr package [33]. The following settings were used: number of permutations = 1000, q-value <0.001, fold change >2. Heatmaps were generated using the gplots package [34].

Least absolute shrinkage and selection operator (LASSO) analyses [35] were performed with R 3.0.2 [32] and the glmnet package [36]. The following settings were used: family = binomial, alpha = 1, lamba = 1 SE from minimum. As described, prior to LASSO analysis, ELISA values for each autoantigen were normalized to the 95^th^ percentile value [23]. Urinalysis results were converted to binary variables: urine red blood cell (URBC) or white blood cell count (WBC) >5 per high-power field (HPF) and urine protein to creatinine ratio >0.2 were considered abnormal. In rare instances where a clinical laboratory result was not available for a specific visit (e.g. contaminated urinalysis), the result from the closest visit in time was carried back or forward. If a clinical laboratory result from another visit was not available, the median value was used. In an effort to generate a model suitable for monitoring renal activity, we removed additional variables that are typically only collected at the initial visit prior to performing LASSO, including SLE diagnostic criteria, APL, anti-Sm, anti-RNP, anti-Ro, and anti-La. This did not significantly impact the performance of the model. The input variables used in the LASSO analysis are listed in Table S1 in Additional file 1.

Based on the training set data, LASSO was used to fit linear models in a stepwise progression along a sequence of values that penalize model complexity (magnitude of the coefficient vector). Cross-validation was used to estimate the best penalty value, and the coefficients at this value were used to create the linear equation shown here:

$$ \mathrm{Score}=-4.193+2.606\left(\mathrm{C}1\mathrm{q}\right)+2.924\left(\mathrm{dsDNA}\right)+0.064\left(\mathrm{W}\mathrm{B}\mathrm{C}\right)-0.013\left(\mathrm{H}\mathrm{g}\mathrm{b}\right)+0.001\left(\mathrm{A}\mathrm{L}\mathrm{C}\right)-0.016\left(\mathrm{C}4\right)-0.012\left(\mathrm{E}\mathrm{S}\mathrm{R}\right)+5.698\left(\mathrm{URBC}\right) $$

This equation was used to calculate each patient’s nephritis score. In the equation, anti-C1q and anti-dsDNA represent normalized serum ELISA values, and the following abbreviations were used: white blood cell count (WBC), hemoglobin (Hgb), absolute lymphocyte count (ALC), complement C4 (C4), erythrocyte sedimentation rate (ESR), and abnormal urine red blood cells (URBC; binary). Coefficients have been rounded to three decimal places.

For ELISA, Prism 6 software (GraphPad Software, Inc., San Diego, CA, USA) was used to perform Mann–Whitney tests (without adjustment for multiple tests), comparing reactivity between patients with and without proliferative nephritis.

## Results

### Autoantigen microarrays identify multiple antibodies associated with pediatric SLE

**Fig. 1:**
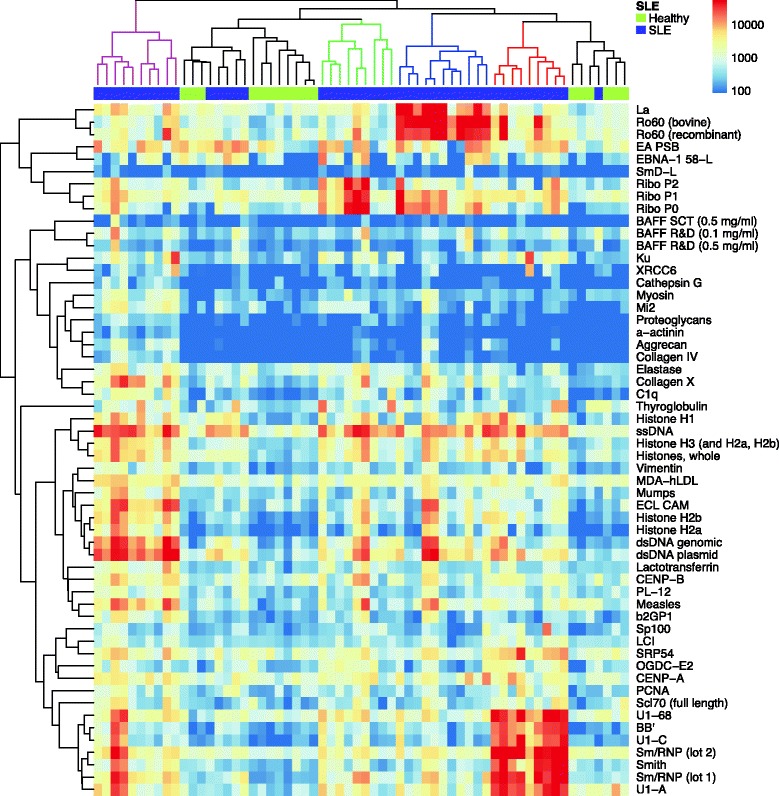
Autoantigen microarrays identify autoantibodies associated with pediatric SLE. Serum samples from 45 new-onset pSLE patients and 17 age-matched healthy controls were used to probe autoantigen microarrays featuring 140 purified or recombinant autoantigens. Cy5-conjugated anti-human IgG antibodies were used as a secondary reagent, a fluorescent microarray scanner was used to image each microarray, and the feature MFIs were used to quantify binding. Significance analysis of microarrays (SAM) was used to identify significant differences in IgG reactivity to the autoantigens between pSLE patients and controls (q-value <0.001, fold change >2). A hierarchically clustered heatmap (complete linkage, unsupervised) of the significant autoantigens is shown, with pSLE patients indicated on the *top bar* in purple and healthy controls in green. Patient clusters are colored red, blue green and magenta in the phylogenetic tree. *IgG* immunoglobulin G, *MFI* median fluorescence intensity, *pSLE* pediatric systemic lupus erythematosus, *SLE* systemic lupus erythematosus

We used serum samples from new-onset pediatric SLE patients (n = 46) and age-matched healthy controls (n = 17) to probe autoantigen microarrays featuring 140 purified or recombinant autoantigens (Table S2 in Additional file 1). To identify autoantibodies that are associated with pSLE, we used significance analysis of microarrays (SAM) to find significant differences in serum IgG reactivity between the pSLE patients and healthy controls (Fig. 1). The analysis identified 50 significant autoantigens, all of which had higher reactivity in pSLE patient serum than in healthy control serum (q-value <0.001, fold change >2), including known serologic markers for SLE (e.g. Ro, La, Smith, dsDNA, histones, RNP). In addition to known markers, our analysis identified candidate autoantigens that, to our knowledge, have not been previously reported in pSLE, including collagen type X, oxoglutarate dehydrogenase complex E2 (OGDC-E2), Speckled 100 kDa (sp100), PL-12, signal recognition particle 54 kDa (SRP54) and BAFF.

Unsupervised hierarchical clustering of the microarray data revealed pSLE patients and healthy controls assorted into multiple distinct groups based on their autoantigen reactivity (Fig. 1). Clusters in the phylogenetic tree are colored to highlight the groups, which predominantly include patients with reactivity to Sm/RNP-related antigens (red), DNA/histone-related antigens (magenta), Ro/La (blue), and RiboP (green). Healthy controls and a small subset of SLE patients had relatively low reactivity to most antigens (black cluster).

### BAFF autoantibodies are associated with active disease in pediatric SLE

**Fig. 2:**
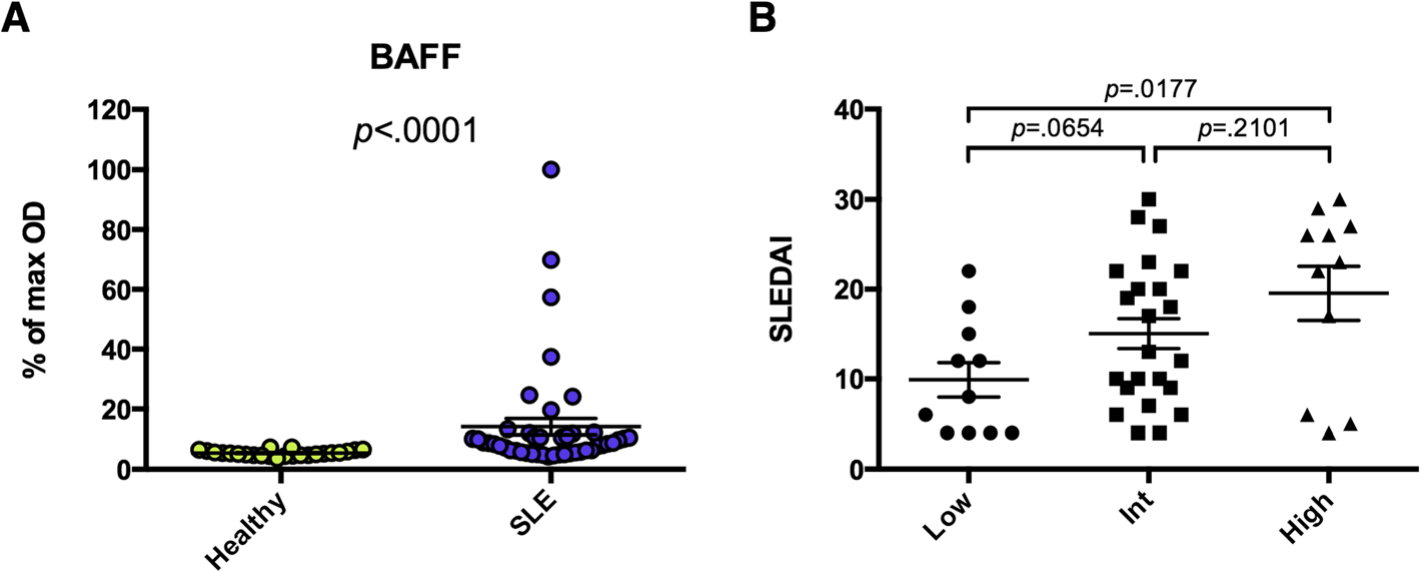
Anti-BAFF is present in a subset of pediatric SLE patients with active disease. **a** Recombinant BAFF was used to coat 96-well ELISA plates, and sera from 45 pSLE patients and 24 age-matched healthy controls were used to probe them in triplicate. HRP-conjugated goat anti-human IgG (heavy and light chain) was used as a secondary reagent. After development with TMB substrate, absorbance was measured at 450 nm, and each sample is shown as a percentage of the maximum absorbance. **b** The pSLE patients were divided into groups based on their serum reactivity to BAFF. The modified SELENA-SLEDAI is shown for the lowest quartile (Low, <5.8), middle two quartiles (Int), and highest quartile (High, >11.0). Mann–Whitney tests were used to compare reactivity between groups (bars show mean ± SEM). *BAFF* B cell-activating factor, *ELISA* enzyme-linked immunosorbent assay, *IgG* immunoglobulin G, *HRP* horseradish peroxidase, *pSLE* pediatric systemic lupus erythematosus, *SELENA-SLEDAI* Safety of Estrogen in Lupus Erythematosus National Assessment-SLE disease activity index, *SLE* systemic lupus erythematosus, *TMB* 3,3′,5,5′-tetramethylbenzidine

Our group previously identified BAFF autoantibodies in the sera of adult SLE patients [20], but BAFF autoantibodies have yet to be reported in pSLE. To validate our microarray finding, we assayed the serum of 45 pSLE patients and 24 healthy controls by indirect serum ELISA, using recombinant BAFF as the antigen. Similar to the microarray, we found that BAFF autoantibodies were at significantly higher levels in the serum of pSLE patients than healthy controls (28/45 pSLE patients had reactivity greater than the maximum healthy control; Fig. 2a). To investigate association of BAFF autoantibodies with disease activity, we divided the pSLE patients into groups based on their serum reactivity to BAFF and compared SLE disease activity index (SLEDAI) between groups (Fig. 2b). Patients with BAFF autoantibody levels in the highest quartile had significantly higher SLEDAI than those in the lowest quartile.

### Autoantigen microarrays identify multiple antibodies associated with proliferative nephritis

**Fig. 3:**
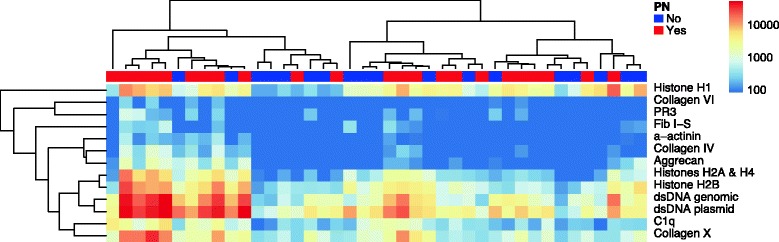
Autoantigen microarrays identify multiple autoantibodies associated with proliferative nephritis in pSLE. Serum samples from 41 new-onset pSLE patients were used to probe autoantigen microarrays featuring 140 purified or recombinant autoantigens. Anti-human IgG antibodies conjugated with Cy5 were used as a secondary reagent, a fluorescent microarray scanner was used to image each microarray, and the feature MFIs were used to quantify binding. Significance analysis of microarrays (SAM) was used to identify significant differences in IgG reactivity to the autoantigens between patients with biopsy-proven class III/IV nephritis and patients without significant evidence of nephritis (q-value <0.06, fold change >2). A hierarchically clustered heatmap of the significant antigens is shown. Patients with biopsy-proven proliferative nephritis are indicated on the *top bar* in red, while patients without nephritis are shown in blue. *IgG* immunoglobulin G, *MFI* median fluorescence intensity, *pSLE* pediatric systemic lupus erythematosus, PN proliferative nephritis

To test whether profiling autoantibodies in the serum of pSLE patients could identify antigen reactivity patterns that are associated with nephritis, we divided the pSLE patients into those with biopsy-proven proliferative nephritis within one year of their initial visit (n = 23), and those with either biopsy-confirmed class II nephritis or no significant evidence of proliferative nephritis (n = 18). We used SAM to identify differences in serum IgG reactivity between the groups (Fig. 3), and found 13 autoantigens with significantly higher reactivity in serum from pSLE patients with proliferative nephritis than those without (q-value <0.001, fold change >2). Anti-dsDNA [3, 10], anti-C1q [10, 37, 38], alpha-actinin [39], and fibrinogen [40, 41] have been previously associated with LN in SLE patients. Additional autoantigens we identified had not been reported previously, including collagens IV and X, aggrecan, and histones H1, H2B and H2A and H4.

### ELISA validation of candidate autoantibodies identified by microarray

**Fig. 4:**
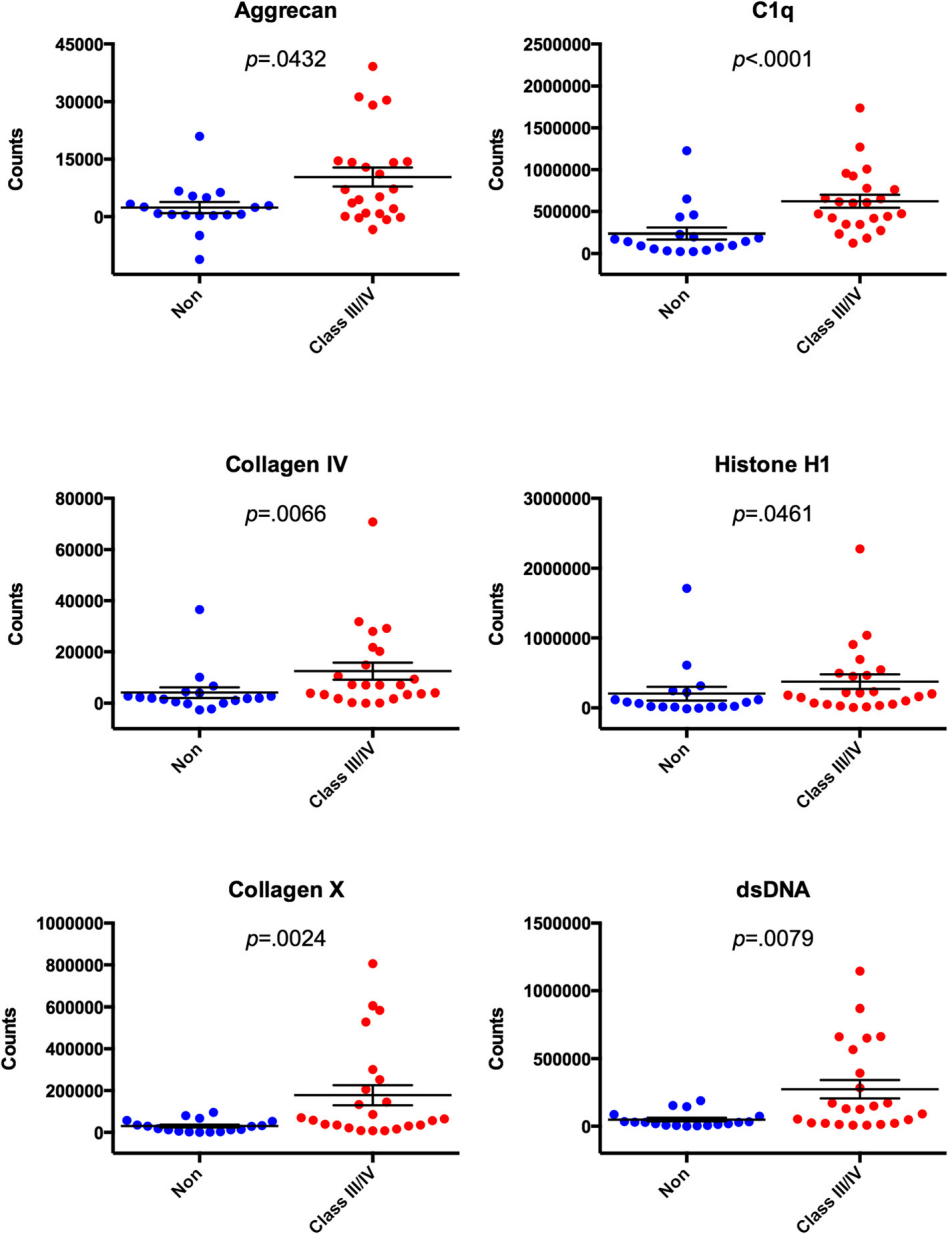
ELISA validation of candidate autoantigens identified by microarray. Significance analysis of microarrays (SAM) identified 13 autoantigens as having significantly different IgG reactivity between pSLE patients with and without proliferative nephritis, of which seven were selected for further validation using indirect serum ELISA. Purified or recombinant forms of the autoantigens shown above were used to coat 96-well ELISA plates. Serum from 42 pSLE patients was used to probe them in duplicate, and europium-labeled anti-human IgG (Fcγ specific) was used as a secondary reagent. Time-resolved fluorescent counts for six of the seven selected autoantigens (minus signal from BSA-coated wells) are shown above. Mann–Whitney tests were used to compare reactivity between groups (bars show mean ± SEM). *BSA* bovine serum albumin, *ELISA* enzyme-linked immunosorbent assay, *IgG* immunoglobulin G, *pSLE* pediatric systemic lupus erythematosus

We selected eight candidate proliferative nephritis-associated autoantigens that were identified by microarray, and utilized indirect serum ELISA to validate our findings. The autoantigens were: dsDNA, complement C1q, collagens IV and X, aggrecan, histone H1, histone H2B, and histones H2A and H4. Serum from the 42 new-onset pSLE patients was assayed for reactivity to each of the autoantigens, and we found a high level of agreement between the microarrays and ELISAs. Six of eight of the autoantigens tested, dsDNA, C1q, collagens IV and X, aggrecan, and histone H1, had significantly higher reactivity in patients with biopsy-proven proliferative nephritis than in those without (Fig. 4). Levels of anti-BAFF, as measured by ELISA, did not show an association with proliferative nephritis (data not shown). These findings confirm anti-dsDNA and anti-C1q are associated with LN in pSLE, identify aggrecan, collagen IV, and collagen X autoantibodies as novel markers of LN in pSLE, and validate autoantigen microarray as a platform for discovery of candidate biomarkers of LN.

### Identification of a pediatric SLE proliferative nephritis predictive signature

**Fig. 5:**
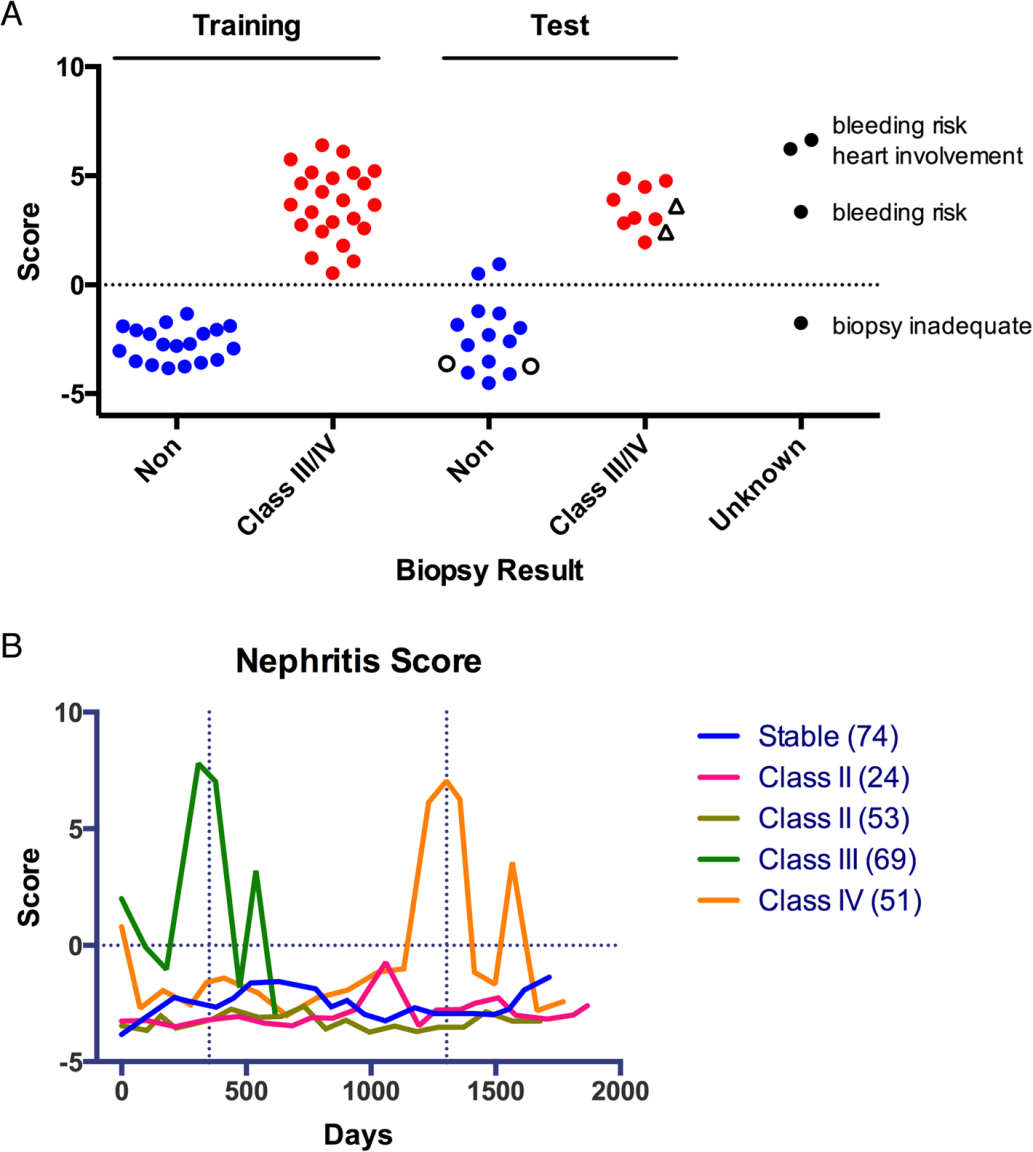
Identification of a pediatric SLE proliferative nephritis predictive signature. **a** Least absolute shrinkage and selection operator (LASSO) was used on a training set of ELISA and clinical measurements from 41 new-onset pSLE patient samples as a variable selection and linear regression method to construct a predictive model of proliferative nephritis. A separate test set of 23 new-onset pSLE patient samples was used to evaluate the performance of the model, and the nephritis scores obtained for each patient with the model are shown. Unfilled *black circles* indicate patients who were biopsied and found to be class II, and unfilled *black triangles* indicate class V nephritis patients. The model was also used to calculate nephritis scores for samples from new-onset pSLE patients who were suspected to have nephritis, but were not confirmed by biopsy (unknown). **b** Five pSLE patients from the test set were selected for longitudinal analysis. ELISA was used to determine each serum sample’s anti-dsDNA and anti-C1q IgG levels, and a chart review was performed to collect all applicable clinical data. The LASSO model based on the training set was used to calculate nephritis scores for each patient at each time point. Vertical dashed lines indicate when biopsies were performed, and the horizontal dashed line indicates the nephritis score cutoff. *ELISA* enzyme-linked immunosorbent assay, *IgG* immunoglobulin G, *pSLE* pediatric systemic lupus erythematosus, *SLE* systemic lupus erythematosus

To construct a predictive model of proliferative nephritis for pSLE patients, we created a training set of ELISA and clinical measurements from the new-onset pSLE patient samples (n = 41) used in the microarray analysis and ELISA validation. We decided to utilize our ELISA results instead of microarray values in order to create a model that could be determined at centers without the requirement of highly specialized microarray equipment. Clinical measurements in the training set included: urinalysis, basic clinical information, SLE diagnostic criteria, and typical SLE laboratory tests (Table S1 in Additional file 1). We used the least absolute shrinkage and selection operator (LASSO) on the training set as a variable selection and linear regression method [35]. LASSO selected the following variables in the model: ELISA measurements of anti-C1q and anti-dsDNA serum IgG, and clinical measurements of absolute lymphocyte count (ALC), white blood cell (WBC) count, blood hemoglobin (Hgb), erythrocyte sedimentation rate (ESR), complement C4 levels, and abnormal urine RBC. We used the predictive model to calculate nephritis scores for each patient sample, which are shown in Fig. 5a. The model successfully partitioned the patients according to whether they did or did not have proliferative nephritis.

To evaluate the performance of the predictive model, we used a separate test set of 23 new-onset pSLE patient samples with biopsy-confirmed class III or IV proliferative nephritis (n = 8), or without significant renal involvement (n = 15), and the nephritis scores obtained for each patient with the model are shown in Fig. 5a. The model correctly categorized patients with proliferative nephritis with an overall accuracy of 91 %. It is important to note that two patients who had enough clinical evidence of nephritis to undergo kidney biopsy, but were found to have class II LN, were correctly assigned negative nephritis scores, highlighting the specificity of the model. Two patients with class V LN were analyzed in parallel with the test set to evaluate the model’s performance on patients with membranous nephritis, and their scores classified them in the proliferative nephritis group. The model was also used to calculate nephritis scores for four ‘unknown’ samples from new-onset pSLE patients who were suspected to have proliferative nephritis, but were not confirmed by biopsy.

### Longitudinal analysis of pediatric SLE predictive signature

To determine whether our model could be used to monitor patients for evidence of proliferative nephritis, we selected five pSLE patients from the test set for longitudinal analysis based on their disease progression. The patients included two who developed biopsy-confirmed proliferative nephritis at 351 days (class III) and 1303 days (class IV) after their initial visit, two age-matched controls who were biopsied and found to have class II LN, and a final subject who had a relatively stable disease course. Samples from 88 patient visits were assayed, with an average of 92 days between visits. ELISA was used to determine each serum sample’s anti-dsDNA and anti-C1q IgG levels, and a chart review was performed to collect all applicable clinical data (Table S1 in Additional file 1). The LASSO model based on the training set was used to calculate nephritis scores for each patient at each time point (Fig. 5b). In the patients who developed proliferative nephritis, nephritis scores increased dramatically and were indicative of proliferative nephritis 43 days (class III) and 71 days (class IV) before kidney biopsies were performed. In the three patients without proliferative nephritis, nephritis scores remained low, indicative of an absence of proliferative nephritis throughout the time course.

## Discussion

In this study, we analyzed a new pSLE cohort using autoantigen microarrays, and identified serum autoantibodies that are associated with clinical manifestations of pSLE. We identified BAFF as a novel autoantigen in pSLE and found that the presence of BAFF autoantibodies was associated with active disease. We found that autoantibodies to collagen IV, collagen X and aggrecan were associated with proliferative nephritis in pSLE. Using autoantibody measurements and clinical information, we created a combined signature capable of accurately identifying pSLE patients with proliferative nephritis.

This is the first report of a new pSLE cohort, which includes a repository of >1087 longitudinal serum samples from 122 pSLE patients, including 71 new-onset patients. Extensive clinical information was collected at each patient visit, covering up to 8 years of clinic visits. An exceptional feature of the cohort is that autoantigen microarray analysis has now been performed on >100 of the patient samples, making in-depth investigation of the role of autoantibodies in pSLE possible.

To our knowledge, this is the first description of using autoantigen microarrays to identify autoantibodies associated with pSLE or predictive of pSLE LN. Our analysis comparing serum IgG reactivity between pSLE patients and healthy controls identified candidate autoantigens that have not been reported in pSLE, including collagen type X, OGDC-E2, sp100, PL-12, SRP54 and BAFF (Fig. 1). Our group previously showed that BAFF autoantibodies are present in the serum of adult SLE patients, and that the autoantibodies were capable of blocking stimulation of the BAFF receptor [20]. In agreement with these findings, we found significantly higher levels of BAFF autoantibodies in the serum of pSLE patients than in healthy controls (Fig. 2a). Further, we found that higher levels of IgG reactivity were significantly associated with elevated SLEDAI scores. This parallels our group’s report that the presence of BAFF autoantibodies was associated with activation of the type I IFN pathway in adult SLE patients [20, 22]. These findings are important in light of the fact that BAFF is the target of a fully human, recombinant monoclonal antibody, belimumab, which was recently approved for the treatment of SLE [42]. Further studies will be required to determine whether SLE patients with BAFF autoantibodies respond differently to belimumab, compared to patients without BAFF autoantibodies.

Unsupervised hierarchical clustering of the serum autoantibodies of pSLE patients and healthy controls revealed multiple distinct patient clusters (Fig. 1). The clusters corresponded to groups in which the predominant autoantibody reactivity was to Sm/RNP-related antigens, DNA/histone-related antigens, Ro/La, or RiboP, or was relatively low to most antigens. This clustering pattern bears similarities to autoantibody clusters previously identified in adult SLE [43, 44] and pSLE [10, 45, 46]. We compared SLEDAI scores and the frequency of proliferative nephritis between pSLE patients within each cluster. We found the low reactivity cluster had lower SLEDAI scores, and the DNA/histone-related antigens cluster had an increased frequency of nephritis, although neither reached statistical significance (data not shown). Other groups have observed a similar association between DNA/histone-related antigen clusters and nephritis or evidence of nephritis [43, 44, 46]. We are currently investigating the association of these clusters with other clinical parameters, including evidence of an association between antibodies to mumps and measles viruses and the DNA/histone cluster.

To identify autoantibodies associated with proliferative nephritis, we compared the serum IgG reactivity of pSLE patients with biopsy-proven proliferative nephritis, and those with either biopsy-confirmed class II nephritis or no significant evidence of proliferative nephritis (Fig. 3). Similar to previous reports, we identified an association between dsDNA [3, 10] and C1q [10, 37, 38] autoantibodies with LN in pSLE (Figs. 3 and 4). Autoantibodies to dsDNA are used in the diagnosis of SLE, fluctuate with disease activity, and are associated with kidney involvement in pSLE [3]. A positive correlation has been observed between glomerulonephritis and C1q autoantibodies in adult SLE [37]. While C1q autoantibodies were found in pSLE patient serum, the same association with LN was not observed in one report [47], but a significant association with LN was observed in more recent studies [10, 48, 49]. Similar to previous reports, we observed an association between LN and autoantibodies to alpha-actinin and fibrinogen [39–41]. Alpha-actinin is an actin-binding protein and member of the spectrin family of proteins. A fraction of dsDNA autoantibodies in the sera of SLE patients also bind alpha-actinin [39]. Presence of these cross-reactive autoantibodies was associated with a higher frequency of glomerulonephritis [39]. Patients in our cohort may also have had cross-reactive autoantibodies, as there was a relatively strong relationship between serum levels of anti-dsDNA and anti-alpha-actinin (R^2^ = 0.594, *p* <0.001).

In addition to autoantigens known to be associated with LN, we found multiple autoantigens that have not been previously associated with proliferative nephritis, including aggrecan, collagen IV and collagen X (Figs. 3 and 4). Aggrecan is the major proteoglycan in articular cartilage, and contributes to its resilience. Aggrecan autoantibodies have been described in SLE, rheumatoid arthritis, systemic sclerosis, Sjögren’s syndrome, and ankylosing spondylitis [50]. Collagen IV is a structural protein that forms networks in the glomerular basement membrane. Autoantibodies to collagen IV have been reported in SLE, although their relation to LN was not discussed [51]. Autoantibodies to collagen IV are more commonly associated with Goodpasture’s disease, a rare autoimmune disease in which autoantibodies damage the lung and kidneys. Collagen X is a homotrimeric short-chain collagen that is primarily expressed by chondrocytes, and is a structural component of articular cartilage. It has previously been described as an autoantigen in type 1 diabetes [52]. Interestingly, we found that autoantibodies to collagen X were also higher in pSLE patients than in healthy controls (Fig. 1). We are currently evaluating whether aggrecan and collagens IV and X are similarly associated with LN in adult SLE.

Pathogenesis of LN is influenced by multiple pathways and factors, including complement, autoantibodies, environment, and genetics [17]. We utilized a stepwise regression method, called LASSO, to generate a predicative model of proliferative nephritis based on multiple clinical and experimental variables (Fig. 5a and Figure S2 in Additional file 1). LASSO constrains the sum of the absolute values of the regression coefficients, shrinking the coefficients of redundant or uninformative variables to zero, resulting in a sparse model. In this way, LASSO models tend to be simplified, interpretable, and efficient. Our model correctly categorized 91 % (21/23) of patients in an independent test set with 100 % sensitivity and 87 % specificity (Fig. 5a). For comparison with other clinical measures, see Figures S1 and S3 in Additional file 1. Our analysis included samples from four new-onset pSLE patients who were suspected to have nephritis, but were not confirmed by biopsy due to inconclusive biopsy, bleeding risk, or heart involvement (Fig. 5a). Our results suggest that the patient with an inconclusive biopsy may not have needed cytotoxic therapy.

Current clinical methods perform poorly at monitoring renal progression, and there is a need for new methods that might allow preemptive management of renal flare. While our longitudinal study had a small sample size, we provide evidence that our nephritis score is predictive of renal flare (Fig. 5b). We found that nephritis scores increased to levels predictive of proliferative nephritis between 43 and 71 days before biopsy. We selected the controls of our longitudinal analysis carefully, including two who were suspected to have nephritis based on clinical measures and observation, but were found to have class II LN on biopsy. These patients would be the most difficult to separate from patients with proliferative nephritis, highlighting the strength of our approach. Interestingly, rebound peaks in the patients’ nephritis scores were observed immediately following biopsy. This appears to be due to the common clinical practice of titrating back cytotoxic treatment following a flare, and suggests that nephritis scores could be used to monitor and avoid similar instances of rebound inflammation. In the future, this approach could be used with a larger cohort to create a similar model to aid clinicians in diagnosing and monitoring proliferative nephritis in pSLE patients.

## Conclusions

Autoantigen microarrays are an ideal experimental platform for identifying novel serum autoantibodies associated with pSLE and clinical manifestations of pSLE, including proliferative nephritis. Using statistical methods, such as LASSO, to integrate data from multiple sources, including serum autoantibody profiles and clinical information, will be crucial to understanding the heterogeneity and complexity of SLE and its clinical manifestations. Our approach of combining serum autoantibody profiles with clinical information represents a potential avenue to enhance care. A similar model developed with a larger multicenter cohort could be used as a regular test for pSLE patients, allowing early detection and preemptive treatment of proliferative nephritis, or in monitoring drug response in clinical trials.

## Additional file

Additional file 1: Table S1. Autoantibody and clinical variables. **Table S2.** Microarray antigens. **Figure S1.** Association of anti-C1q and anti-dsDNA, as well as other clinical measures with proliferative nephritis. **Figure S2.** Cross-validated deviance of LASSO. **Figure S3.** Receiver operating characteristic (ROC) analysis of clinical measures.

## Abbreviations

ALC: absolute lymphocyte count
ANA: antinuclear antibody
APL: antiphospholipid
BAFF: B cell-activating factor
BSA: bovine serum albumin
C4: complement C4
DELFIA: dissociation-enhanced lanthanide fluoroimmunoassay
ELISA: enzyme-linked immunosorbent assay
ESR: erythrocyte sedimentation rate
FCS: fetal calf serum
Hgb: hemoglobin
HPF: high-power field
IFN: interferon
IgG: immunoglobulin G
LASSO: least absolute shrinkage and selection operator
LN: lupus nephritis
MFI: median fluorescence intensity
OGDC-E2: oxoglutarate dehydrogenase complex E2
PBS(T): phosphate-buffered saline (with Tween 20)
pSLE: pediatric systemic lupus erythematosus
RNP: ribonucleoproteins
SAM: significance analysis of microarrays
SCID: severe combined immunodeficiency
SLE: systemic lupus erythematosus
SLEDAI: SLE disease activity index
sp100: Speckled 100 kDa
SRP54: signal recognition particle 54 kDa
URBC: urine red blood cells
WBC: white blood cell count
