# Supplementary material for: Autoantigen microarrays reveal autoantibodies associated with proliferative nephritis and active disease in pediatric systemic lupus erythematosus
Source: Arthritis Res Ther. 2015 Jun 17;17(1):162. doi: 10.1186/s13075-015-0682-6 (PMC4493823; doi:10.1186/s13075-015-0682-6)
Supplement: Additional file 1: Table S1. — Autoantibody and clinical variables. Table S2. Microarray antigens. Figure S1. Association of anti-C1q and anti-dsDNA, as well as other clinical measures with proliferative nephritis. Figure S2. Cross-validated deviance of LASSO. Figure S3. Receiver operating characteristic (ROC) analysis of clinical measures. [file 13075_2015_682_MOESM1_ESM.pdf]

Category	Variable
**ELISA measurements**	
	Histones H1, H2B, and H2A & H4
	Complement C1q*
	Collagens IV and X
	Aggrecan
	dsDNA*
**Clinical**	
	Gender
	Age at diagnosis and at sample
	Modified SELENA-SLEDAI
	Physician's global assessment (PGA)#
	White blood cell count (WBC)*
	Hemoglobin*
	Platelets
	Absolute lymphocyte count (ALC)*
	Creatinine
	Complement C3 and C4*
	Erythrocyte sedimentation rate (ESR)*
**Urinalysis**	
	Protein:creatinine ratio
	Red blood cells (RBC)*
	White blood cells (WBC)
	Granular/cellular casts
**Autoantibodies**	
	DNA
	Smith#
	Ribonucleoprotein (RNP)#
	Ro & La#
	Antiphospholipid (APL)#
**Lupus Criteria#**	
	Malar rash
	Discoid rash
	Photosensitivity
	Oral ulcers
	Arthritis
	Serositis
	Renal
	Central nervous system (CNS)
	Hematological
	Autoantibodies
	Antinuclear antibodies (ANA)

Supplemental Table S1. Autoantibody and clinical variables.
* Variables included in the training set model
# Variables removed prior to LASSO analysis

Antigen	Vendor	Catalog number	Description
a-actinin	Sigma	A9776	Chicken gizzard
Actin	Molecular Probes	A12375	Rabbit muscle
Aggrecan	Sigma	A1960	Bovine articular cartilage
aKGDH	Sigma	K1502	Porcine heart
Aldolase	Sigma	A2714	Rabbit muscle
Annexin V-d	Diarect	19101	Recombinant human, His-tagged
Annexin V-s	Sigma	A9460	Human placenta
Azurocidin	The Binding Site	BH236.X	Human neutrophils
b2GP1	Diarect	A14901	Recombinant human, His-tagged
BAFF	R&D	2149-BF-010	BLyS/TNFSF13B
BAFF	Stem Cell	02517	Recombinant human
BB'	Diarect	13300	Recombinant human, His-tagged
BCOADC-E2	Diarect	Test lot 1201/2	Recombinant human, His-tagged
BPI	Diarect	Test lot 1352	From purified human neutrophils
BPI	Arotec	ATB01-02	From purified human neutrophils
Ro 60/SS-A (bovine)	Diarect	A15501	Bovine
C1q	Biodesign	A90150H	Human serum
Cardiolipin 0	Sigma	C0563	Bovine heart
Cardiolipin 1	Sigma	C1649	Bovine heart
Catalase	Sigma	C3556	Human erythrocytes
Cathespin G	Arotec	ATC01-02	Human neutrophils
CENP-A	Diarect	A16901	Recombinant human, His-tagged
CENP-B	Diarect	A12501	Recombinant human, His-tagged
Collagen II	Sigma	C1188	Human placenta
Collagen IV	Sigma	C5533	Human placenta
Collagen IX	Sigma	C3657	Human placenta
Collagen VI	Sigma	C7521	Human placenta
Collagen VIII	Sigma	C7774	Human placenta
Collagen X	Sigma	C4407	Human placenta
Artemis (DCLRE1C)	Abnova	H00064421-P01	Recombinant human
Diphtheria Toxin	Sigma	D0564	Corynebacterium diphtheriae
dsDNA genomic	Sigma	D1626	From salmon testes
dsDNA plasmid	Diarect	12301	E.Coli
Desmoglein 1 (DSG1)	Abnova	H00001828-P01	Recombinant human
Desmoglein 4 (DSG4)	Abnova	H00147409-Q01	Recombinant human
EA-D	Biodesign	R18740	Recombinant
EA	ProspecBio	EBV-272	Recombinant
EBNA-1 35 D isomer	Sigma	aa 35-58	Sequence: GGDNHGRGRGRGRGRGGGRPGAPG
EBNA-1 35 L isomer	Sigma	aa 35-58	Sequence: GGDNHGRGRGRGRGRGGGRPGAPG
EBNA-1 398 D isomer	Sigma	aa 398-412	Sequence: PPPGRRPFFHPVGEA
EBNA-1 398 L isomer	Sigma	aa 398-412	Sequence: PPPGRRPFFHPVGEA
EBNA-1 58 D isomer	Sigma	aa 58-72	Sequence: GGSGSGPRHRDGVRR
EBNA-1 58 L isomer	Sigma	aa 58-72	Sequence: GGSGSGPRHRDGVRR
EBNA-1	Biodesign	R57523	Recombinant
EBNA-1	ProspecBio	EBV-276	Recombinant, his-tagged
Entactin-Collagen IV-Laminin Cell Attachment Matrix (ECL-CAM)	USBiological	E0275	Engelbreth-Holm-Swarm mouse tumor
Elastase	Arotec	ATE01	Human neutrophils
Enolase	Sigma	E0379	Rabbit muscle

Antigen	Vendor	Catalog number	Description
Fib I	Sigma	F3879	Human plasma
Fib I-S	Sigma	F8630	Bovine plasma
Fib IV	Sigma	F4753	Bovine plasma
GBM dissociated	Diarect	A16801	Recombinant human, His-tagged
GBM undissociated	Diarect	A15901	Recombinant human, His-tagged
gp210	Diarect	A19001	Recombinant human, His-tagged
GRP78	Stressgen	SPP-765	Recombinant hamster
Hepatitis B Surface Antigen (HBSAg)	Meridian Life Sciences	R36100	Human plasma
heparan sulfate	Sigma	H7640	Bovine kidney
hHDL	Biomedical	80P-HD101	Human plasma
Human IgG	Jackson	009-000-003	Human
Human IgM	Jackson	009-000-012	Human
Histones, whole	Immunovision	HIS-1000	Bovine Tissue
Histone H1	Immunovision	HIS-1001	Bovine Tissue
Histone H2a & H4	Immunovision	HIS-1002	Bovine thymus
Histone H2b	Immunovision	HIS-1003	Bovine Tissue
Histones H3	Immunovision	HIS-1004	Bovine Tissue
HSP25	Stressgen	NSP-510	Recombinant murine
HSP47	Stressgen	NSP-535	Recombinant human
HSP60	Stressgen	NSP-540	Recombinant human
HSP70	Stressgen	NSP-555	Recombinant human
HSP90	Stressgen	SPP-770	Human, from HeLa cells
Insulin	Sigma	I0908	Recombinant human
Intrinsic factor	Diarect	A12901	Recombinant human, His-tagged
Jo-1 (Histidyl-tRNA synthetase)	Diarect	A12901	Recombinant human, His-tagged
Ku (p70/p80)	Diarect	A17301	Recombinant human, His-tagged
La/SSB	Diarect	A12801	Recombinant human, His-tagged
Lactotransferrin	Abcam	ab78526	Human breast milk
Laminin	Sigma	L2020	EHS murine sarcoma basement membrane
Liver Cytosol Type-1 Antigen (LC1)	Diarect	13700	Recombinant human, His-tagged
DNA ligase 4 (LIG4)	Abnova	H00003981-Q01	Recombinant human
Lipoprotein lipase	Sigma	L9656	Pseudomonas species
Cytochrome P450 2D6 (LKM1)	Diarect	A13501	Recombinant human
Lysozyme	Calbiochem	440345	Human neutrophils
M2	Diarect	A18001	Recombinant human, His-tagged
MAGE-B2	N/A	N/A	Provided by Deborah McCurdy, MD
MDA-hLDL	Academy BioMedical	20P-MD-L110	From human plasma
Rubeola (Measles)	Biodesign	R14120	Edmonston strain
Mi2	Diarect	A18101	Recombinant human, His-tagged
MMP-9	Arotec	ATM03-02	Human neutrophils
MPO	Diarect	18501	Native human
MPO	Immunovision	MPO-3000	Human promyelocytic cell line
Mumps	Biodesign	R02602	BSC-1, strain Enders
Myosin	Sigma	M1636	Rabbit muscle
NGAL	Arotec	ATN01-02	Human neutrophils
XLF (NHEJ1)	Abnova	H00079840-P01	Recombinant human

Antigen	Vendor	Catalog number	Description
NR2A D isomer	Sigma Genosys	Custom	Sequence: SVSYDDWDYSLEARV
NR2A L isomer	Sigma Genosys	Custom	Sequence: SVSYDDWDYSLEARV
Nucleoporin p62 (Nup62)	Diarect	19401	Recombinant human, His-tagged
OGDC-E2	Diarect	Test lot 1196/2	Recombinant human, His-tagged
Ox-hLDL	Academy Biomedical	Ox-L110	From human plasma
EBV P18	ProspecBio	EBV-273	Recombinant
Parietal cell Ag	Arotec	ATP01-04	Porcine gastric mucosa
PCNA	Diarect	15401	baculovirus/insect cell expression
PDC-E2	Diarect	Test lot 359/1	Recombinant human, His-tagged
Pyruvate dehydrogenase (PDH)	Sigma	P7032	Porcine heart
Pertussis Toxin	Sigma	P7208	Bordetella pertussis
PL-12	Diarect	A15701	Recombinant human, His-tagged
PL-7	Diarect	A15601	Recombinant human, His-tagged
PM/Scl 100	Diarect	A16001	Recombinant human, His-tagged
PM/Scl75	Diarect	Test lot 1137/2	Test lot, data not provided
PR3	Diarect	18601	Native human
PR3	Immunovision	PR3-3100	Human promyelocytic cell line
DNA PKcs (PRKDC)	Abnova	H00005591-Q01	Recombinant human
Proteoglycans	Sigma	P5864	Bovine nasal septum
Ribosomal phosphoprotein P0 (Ribo P0)	Diarect	A14101	Recombinant human, His-tagged
Ribosomal phosphoprotein P1 (Ribo P1)	Diarect	Test lot 1193	Recombinant human, His-tagged
Ribosomal phosphoprotein P2 (Ribo P2)	Diarect	Test lot 1179	Recombinant human, His-tagged
Ro 52	Diarect	A12701	Recombinant human, His-tagged
Ro 60/SS-A (recombinant)	Diarect	A17401	Recombinant human, His-tagged
Rubella	Biodesign	R9A123	Vero Cells, strain HPV-77
Scl70 (full length)	Diarect	A12401	Recombinant human, His-tagged
Scl70 (truncated)	Diarect	A14501	Recombinant human, His-tagged
Sm/RNP	Immunovision	SRC-3000	Rabbit and calf thymus
Sm/RNP	Immunovision	SRC-3000	Rabbit and calf thymus
SmD D isomer	Sigma Genosys	Custom	Sequence: AGRGRGRGRGRGRGRGRGRGGPRR
SmD L isomer	Sigma Genosys	Custom	Sequence: AGRGRGRGRGRGRGRGRGRGGPRR
Smith	Immunovision	SMA-3000	Bovine spleen and/or thymus
Sp100	Diarect	A18901	Recombinant human, His-tagged
Signal recognition particle 54 (SRP54)	Diarect	A18401	Recombinant human, His-tagged
ssDNA	Sigma	D8661	Calf thymus
Tetanus Toxin	Sigma	T3194	Clostridium tetani
Thyroglobulin	Diarect	A12201	Native human
TPO (Thyroperoxidase)	Diarect	A12101	Recombinant human, His-tagged
Tissue Transglutaminase	Diarect	A15200	Recombinant human, His-tagged

Antigen	Vendor	Catalog number	Description
U1-snRNP 68 protein	Diarect	A13001	Recombinant human, His-tagged
U1-snRNP A protein	Diarect	A13101	Recombinant human, His-tagged
U1-snRNP C protein	Diarect	A13201	Recombinant human, His-tagged
EBV VCA	Biodesign	R02150	VCA gp125, Burkitt's lymphoma (not recombinant)
Vimentin	Sigma	V4383	Bovine lens
XRCC4	Abnova	H00007518-P01	Recombinant human
Ku80 (XRCC5)	Abnova	H00007520-P01	Recombinant human
XRCC6 (Ku70)	Abnova	H00002547-P01	Recombinant human
Goat Anti-Human IgG, Fcg specific	Jackson	109-005-098	min X Bov, Hrs, Ms Sr Prot
Rabbit Anti-Human IgG + IgM (H+L)	Jackson	309-005-107	min X Ms Sr Prot
Rabbit Anti-Human IgM, Fc5mu Specific	Jackson	309-005-095	min X Ms Sr Prot

Supplemental Table S2. Microarray antigens.

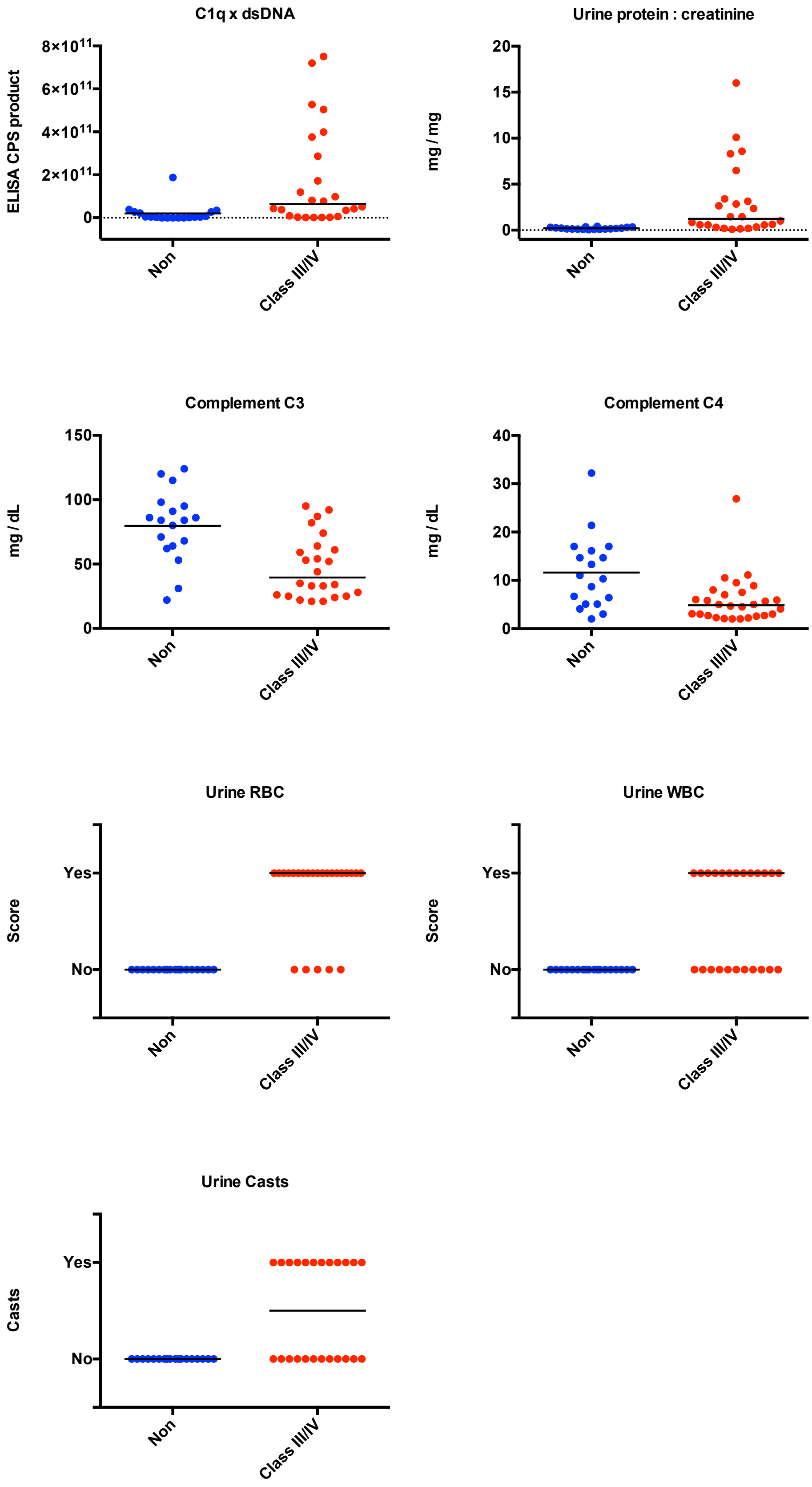
C1q x dsDNA
ELISA CPS product
8×10^11
6×10^11
4×10^11
2×10^11
0
Non
Class III/IV
Urine protein : creatinine
mg / mg
20
15
10
5
0
Non
Class III/IV
Complement C3
mg / dL
150
100
50
0
Non
Class III/IV
Complement C4
mg / dL
40
30
20
10
0
Non
Class III/IV
Urine RBC
Score
Yes
No
Non
Class III/IV
Urine WBC
Score
Yes
No
Non
Class III/IV
Urine Casts
Casts
Yes
No
Non
Class III/IV

Supplemental Figure S1. Association of anti-C1q and anti-dsDNA, as well as other clinical measures with proliferative nephritis. Sera from new-onset pSLE patients (n=42) were divided into to groups based on whether they had biopsy-confirmed proliferative nephritis (n=24), or no significant evidence of nephritis (n=18). Bars represent the medians of each group. C1q and dsDNA counts (minus BSA counts) were multiplied to give the ELISA CPS product. Urine RBC or WBC > 5 per high power field (HPF) were considered abnormal.

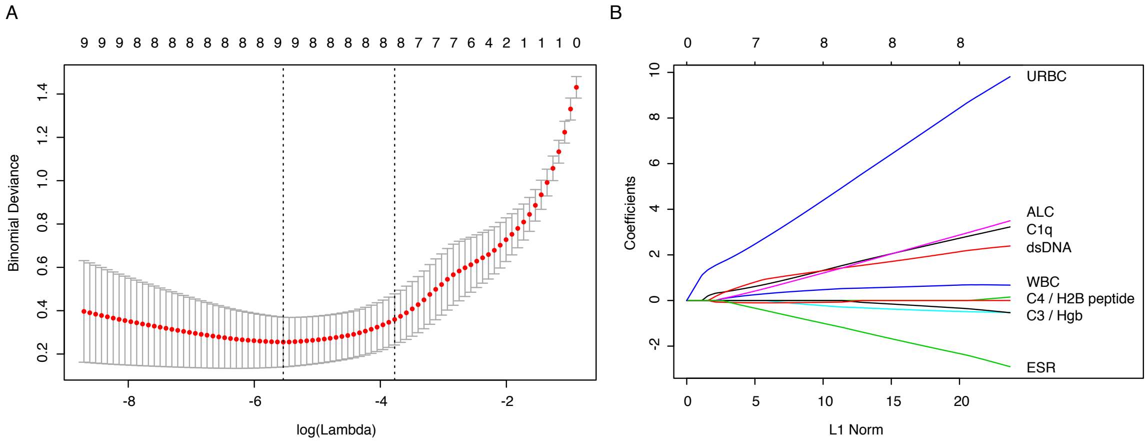

Supplemental Figure S2. Cross-validated deviance of LASSO. (A) Plot of cross-validation deviance along the Lamda sequence. The tuning parameter Lambda controls the overall strength of the penalty for model complexity. The top y-axis indicates the number of nonzero coefficients. The left dashed line indicates the point with minimum cross-validation deviance, and the right dashed line indicates the point one standard deviation above the minimum. The coefficients at the minimum plus one standard deviation were used to create the model. The error bars represent standard deviation. (B) Plot showing nonzero coefficients as a function of the L1 Norm of the entire coefficient vector. The L1 Norm represents the magnitude of the combined coefficient vector. Variables with the greatest magnitude at low L1 Norm have the greatest impact on the nephritis score. Variable names are shown to the right of each line. Similar to A, the top y-axis indicates the number of nonzero coefficients.

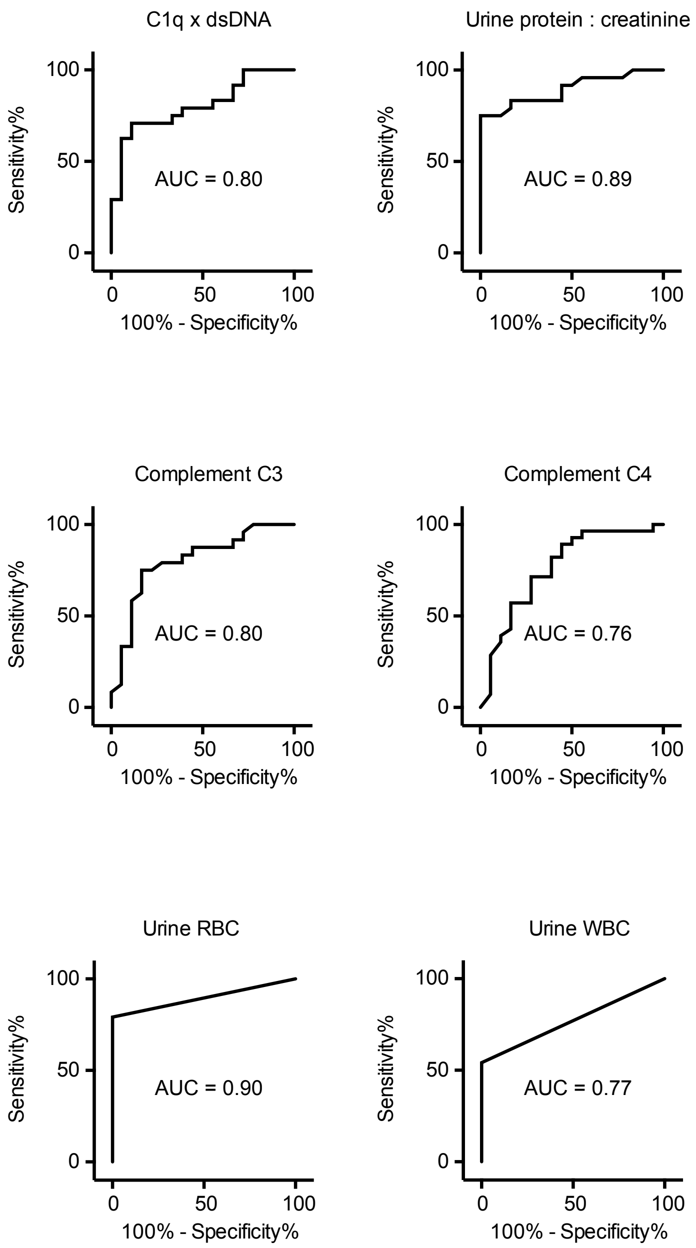

Supplemental Figure S3. Receiver Operating Characteristic (ROC) analysis of clinical measures. New-onset pSLE patients (n=42) were divided into groups based on whether they had biopsy-confirmed proliferative nephritis (n=24), or no significant evidence of nephritis (n=18). ROC curves are shown for combined anti-C1q and anti-dsDNA as well as other clinical measures. Area Under the Curve (AUC) values are shown for each variable. For comparison, the LASSO model had AUC of 1.00.
